# Addictive Potential of Methylphenidate: A Systematic Review of Human and Preclinical Behavioral, Clinical and Neurobiological Evidence

**DOI:** 10.3390/ph19091409

**Published:** 2026-09-06

**Authors:** Rafał Bieś, Adam Chabrzyk, Katarzyna Zborowska, Krzysztof Krysta, Marek Krzystanek

**Affiliations:** 1 Department and Clinic of Psychiatric Rehabilitation, Faculty of Medical Sciences, Doctoral School, Medical University of Silesia, Ziołowa 45/47, 40-635 Katowice, Poland; 2 Department of Clinical Psychiatry and Psychology, Penta Hospitals International, 1000-Lecia 13, 32-300 Olkusz, Poland; adam.chabrzyk@pentahospitals.pl; 3 Department of Clinical and Community Psychiatry and Psychology, Collegium Medicum, WSB University, Cieplaka 1c, 41-300 Dąbrowa Górnicza, Poland; kzborowska@wsb.edu.pl (K.Z.); marek.krzystanek@wsb.edu.pl (M.K.); 4 Department and Clinic of Psychiatric Rehabilitation, Department of Psychiatry and Psychotherapy, Faculty of Medical Sciences, Medical University of Silesia, Ziołowa 45/47, 40-635 Katowice, Poland; ochojec@gmail.com

**Keywords:** methylphenidate, ADHD, substance use disorder, drug misuse, tolerance, craving, withdrawal, psychostimulants, addiction risk

## Abstract

**Background/Objectives**: Methylphenidate is one of the most widely prescribed psychostimulants for attention-deficit/hyperactivity disorder, with increasing global use. Its pharmacological action on dopaminergic pathways raises concerns regarding its addictive potential. This systematic review aims to synthesize current evidence on the addictive properties of methylphenidate, including misuse, abuse, tolerance, craving, and withdrawal symptoms. **Methods**: This systematic review was conducted in accordance with PRISMA guidelines and registered in the PROSPERO database under registration number CRD420261303803. Literature searches were performed in PubMed, Web of Science, and Google Scholar without time restrictions. Studies assessing behavioral, subjective, or neurobiological indicators of methylphenidate addiction were included. Eligible designs comprised randomized controlled trials, observational studies, neuroimaging studies, case reports, and relevant preclinical models. Study quality was assessed using the EPHPP tool. A total of 35 studies were included in the qualitative synthesis. **Results**: The evidence demonstrated that methylphenidate produced measurable reinforcing effects and subjective drug liking, which were found to be closely related to craving and to depend on dose, pharmacokinetics, and route of administration. Rapid increases in brain dopamine levels, particularly following non-oral or high-dose administration, were associated with higher abuse potential. Misuse was observed to be more common among individuals without ADHD and was often motivated by cognitive enhancement rather than euphoria. In contrast, appropriately monitored therapeutic use was associated with a low observed risk of substance use disorders. Pharmacodynamic tolerance was identified in a subset of patients, whereas a classical withdrawal syndrome was not consistently observed and was generally limited to symptom recurrence. **Conclusions**: Methylphenidate exhibits measurable addictive potential. However, its clinical significance is context-dependent. Under therapeutic conditions, the risk of addiction is low, whereas it increases substantially with non-medical use, high doses, and alternative routes of administration. These findings support careful patient selection, preference for extended-release formulations, and monitoring of misuse risk.

## 1. Introduction

Methylphenidate (MPH) is one of the most commonly used psychostimulant drugs worldwide [1]. Its use has been steadily increasing in recent years, due to the growing number of ADHD diagnoses. Over time, a clear rise in the use of stimulant medications has been observed. Data from 2011 to 2021 indicate that prescription rates increased by about 70 percent during this period [2]. Greater availability of MPH has also led to more reports of its inappropriate use, including obtaining the drug without a prescription. It also involves changing doses without medical supervision and using them for non-medical purposes [3].

MPH is an inhibitor of dopamine and norepinephrine transporters. Its action increases dopamine levels in the striatum and enhances catecholaminergic signaling in the central nervous system [4]. The drug is available in different pharmacokinetic formulations. These include immediate-release and extended-release forms. This allows the dosing regimen to be adjusted to the patient’s symptom profile [5]. At therapeutic doses, MPH has a favorable safety profile. However, its use is associated with several risks [6]. The mechanism that increases dopamine availability in reward-related brain structures contributes to its addictive potential. This risk is particularly relevant in cases of non-medical use. It is also higher when large doses are used, when prescribed doses are exceeded, or when the drug is administered by routes that produce a rapid increase in brain concentration [7].

The term “addictive symptoms” refers to a set of clinical manifestations associated with the development of a substance use disorder. These include tolerance, craving, withdrawal symptoms, and persistent or compulsive patterns of use [8]. Tolerance is defined as the need to use increasing doses to achieve the same effect [9]. Craving refers to an intense and difficult-to-control urge to take the substance [10]. Withdrawal symptoms occur after abrupt discontinuation of use. Compulsive use is a key feature of addiction. It is characterized by continued use despite awareness of harm and loss of control over substance intake [11]. The literature also distinguishes between misuse and substance use disorder. Misuse refers to the use of a drug in a manner inconsistent with medical recommendations. This includes higher doses, alternative routes of administration, or recreational use. However, it does not meet the full diagnostic criteria for a disorder. Substance use disorder is a diagnosable condition according to DSM-5 criteria. It requires the presence of at least two out of eleven criteria. These include impaired control, risky patterns of use, social consequences, and physiological factors such as tolerance and withdrawal [12].

MPH increases dopamine levels in the mesolimbic system by blocking the dopamine transporter. This leads to enhanced synaptic dopaminergic activity that is relevant for reward mechanisms [13]. Although its mechanism of action is similar to that of many drugs such as amphetamine and cocaine, a key difference lies in pharmacokinetics. Most stimulant drugs enter and act in the brain very rapidly. This produces a fast and short-lasting high. Orally administered MPH shows a slower increase in brain concentration and a longer duration of action. This reduces its immediate euphoric potential [14]. For this reason, the risk of MPH abuse increases significantly when the drug is administered by non-oral routes. These include intranasal and intravenous use. Such routes accelerate the rise in plasma and brain concentrations. This makes its pharmacokinetics more similar to fast-acting stimulants and increases sensitivity to its rewarding effects [15].

For years, there has been an ongoing debate about the extent to which MPH resembles amphetamine in its effects on the central nervous system and its addictive potential. Both substances are classified as stimulants and have a clear impact on catecholaminergic systems. These controversies arise mainly from the fact that, despite similar clinical effects, the neurobiological mechanisms of MPH and amphetamine differ substantially. Amphetamine acts primarily as a dopamine releaser. It reverses the direction of dopamine transporter function and increases neurotransmitter release from presynaptic terminals [16]. In contrast, MPH mainly functions as an inhibitor of dopamine and norepinephrine reuptake [17]. These differences translate into distinct pharmacodynamic profiles and different euphoric potential. At the same time, the overlap in pharmacological effects forms the basis of the debate on drug misuse. As a result, MPH remains at the center of this discussion. On one hand, it is a well-documented and effective treatment for ADHD in clinical settings. On the other hand, its pharmacological similarity to amphetamine raises concerns about the risk of misuse. This is particularly relevant at higher doses, when immediate-release formulations are used, and outside controlled therapeutic settings.

Despite the widespread clinical use of methylphenidate, important uncertainties remain regarding its addictive potential across different patterns of exposure. Previous studies have reported both potentially protective and potentially adverse associations between methylphenidate treatment and later substance-related problems, while the evidence regarding addiction-related symptoms during prescribed use remains inconsistent [18]. Moreover, the available evidence is dispersed across studies examining different dimensions of addictive potential, including subjective drug liking and reinforcement, craving, tolerance, withdrawal, misuse, and abuse, with substantial variation in dose, formulation, pharmacokinetic profile, and route of administration. This fragmentation makes it difficult to determine how these factors collectively influence the addictive potential of methylphenidate.

Therefore, the present systematic review was undertaken to integrate the available human behavioral, clinical, and neurobiological evidence concerning the addictive potential of methylphenidate. In particular, this review aims to characterize the evidence for reinforcing effects, craving, tolerance, withdrawal, misuse, and abuse; to examine how dose, formulation, pharmacokinetics, and route of administration modify these effects; and to distinguish the risks associated with appropriately monitored therapeutic use from those associated with non-medical use. By bringing these complementary lines of evidence together, this review seeks to provide a more integrated assessment of the circumstances under which methylphenidate may exhibit addictive properties and to identify important gaps in the current evidence base.

## 2. Materials and Methods

This systematic review was conducted in accordance with PRISMA guidelines (Preferred Reporting Items for Systematic Reviews and Meta-Analyses) [19]. The review was registered in the PROSPERO database under registration number CRD420261303803. The Supporting Information can be downloaded at: https://zenodo.org/records/20323061, accessed on 6 September 2026, title: PRISMA 2020 Checklist, DOI: https://doi.org/10.5281/zenodo.20323061.

### 2.1. Inclusion and Exclusion Criteria

Studies were included if they assessed the addictive potential of MPH in humans or in preclinical models. Eligible studies examined at least one of the following domains: behavioral, subjective, or neurobiological indicators of reward, such as drug liking or craving, as well as tolerance, withdrawal symptoms, misuse, or abuse. Randomized and observational studies were included. These involved either healthy volunteers or individuals with ADHD. Neuroimaging studies, case series, single case reports, and preclinical studies were also considered. Preclinical studies were included only when they provided mechanistic insights relevant to the interpretation of clinical findings. Studies addressing both therapeutic use and non-medical use of MPH were eligible. This included alternative routes of administration and exposure patterns associated with misuse.

Studies were excluded if they focused solely on the efficacy of ADHD treatment without addressing addiction-related symptoms, tolerance, or problematic use. Articles describing other stimulants were also excluded if data specific to MPH could not be clearly separated. Opinion-based papers without empirical data were excluded. Conference abstracts and publications not available in English were also excluded. Studies were not included if the reported symptoms could be clearly explained by other neurological or psychiatric conditions, and if there was no evidence supporting a causal link with MPH exposure. Importantly, the presence of empirical or experimental data alone was not sufficient for inclusion. Studies were eligible only when the reported data directly addressed at least one of the predefined addiction-related outcomes relevant to the research question. Studies containing experimental data on methylphenidate were excluded when their primary outcomes concerned therapeutic efficacy, general pharmacological effects, cognitive performance, or other clinical or physiological effects without assessing addictive potential, reinforcement, craving, tolerance, withdrawal, misuse, or abuse. No publication-year restriction was applied. Studies were excluded primarily when they lacked data on addiction-related symptoms or other predefined addiction-related outcomes, when methylphenidate exposure was unclear or insufficiently characterized, or when the study focused exclusively on therapeutic efficacy without addressing the outcomes relevant to the present review.

### 2.2. Search Strategy

A literature search was conducted across PubMed, Web of Science, and Google Scholar without time restrictions. Google Scholar was used as a complementary source to increase the sensitivity of the search and identify potentially relevant publications not retrieved through the other bibliographic databases. Records identified through all sources were subsequently assessed according to the predefined inclusion and exclusion criteria, and relevance ranking in Google Scholar was not used as a criterion for study inclusion.

The following keywords were used to identify relevant studies: Methylphenidate, Ritalin, Concerta, Focalin, and Medikinet. For topics related to misuse, addiction, and behavioral problems, the search also included terms such as Substance-Related Disorders, Drug Abuse, Substance Use Disorder, Addictive, Prescription Drug Misuse, Self Medication, abuse, misuse, dependence, addiction, nonmedical use, off-label use, diversion, recreational use, illegal use, illicit use, street use, overdose, toxic use, drug seeking, doctor shopping, stimulant abuse, stimulant dependence, shared medication, enhancement use, cognitive enhancement, study drug use, exam performance, intoxication, withdrawal symptoms, dependence syndrome, tolerance, and compulsive use. These terms were combined using Boolean operators (AND/OR) in different configurations according to the search interface of each database. Initial screening was based on titles and abstracts, and records were assessed according to the predefined inclusion and exclusion criteria. Reference lists of selected articles were also reviewed to identify additional studies that may have been missed during the database search. Studies most relevant to the topic were selected. Reference lists of selected articles were also reviewed to identify additional studies that may have been missed during the database search.

### 2.3. Study Selection

**Figure 1:**
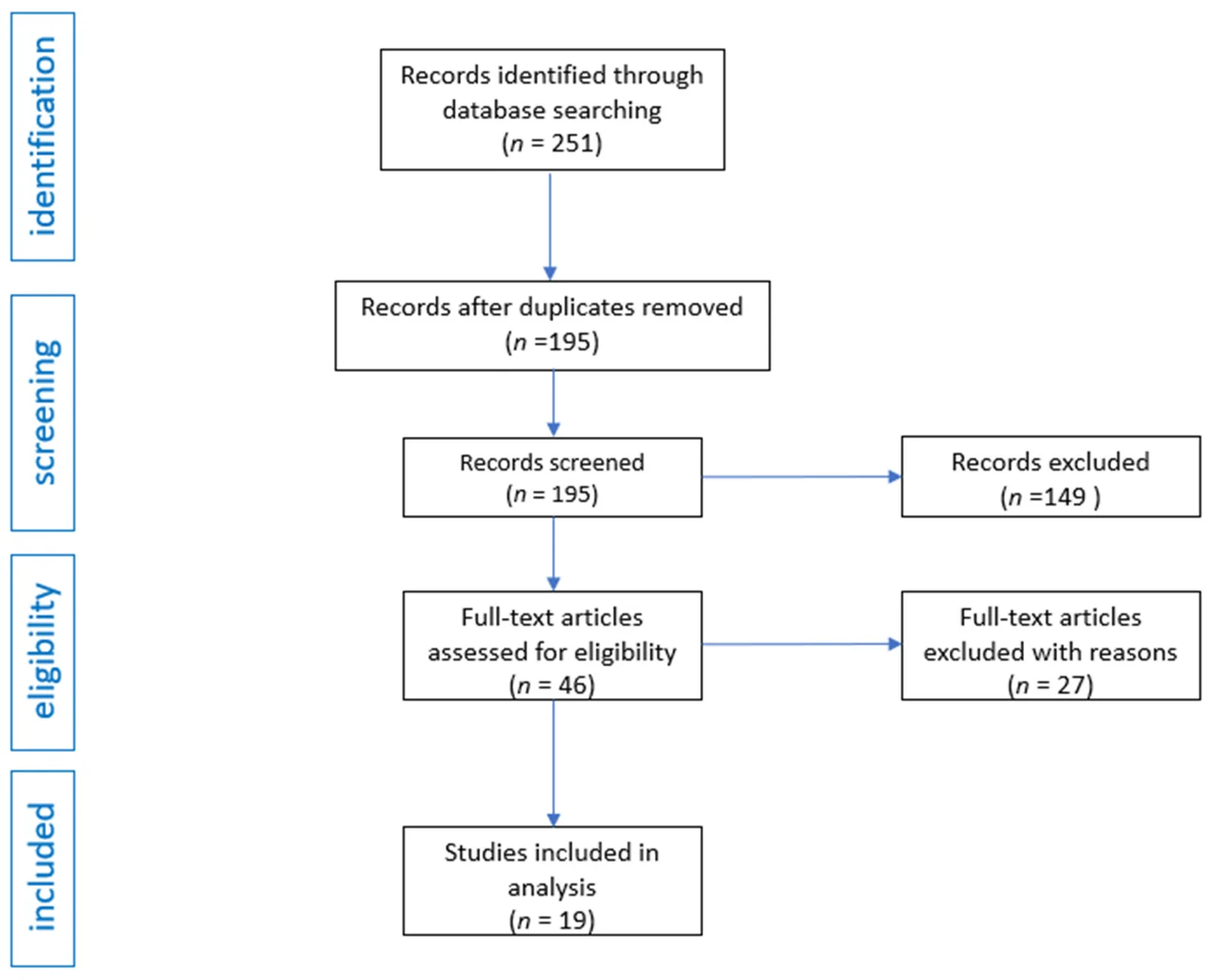
Flow diagram of studies analysis and selection for review.

A total of 1027 records were identified through database searching. After removing duplicates, 753 unique publications were included for further analysis. These were screened based on titles and abstracts. During the initial screening, 640 records were excluded as they did not meet the predefined inclusion criteria. Full texts of 113 articles were then assessed for eligibility. After full-text evaluation, 78 publications were excluded. The main reasons were lack of data on addiction-related symptoms, unclear exposure to methylphenidate, or a sole focus on therapeutic efficacy without reference to the analyzed outcomes. Finally, 35 studies meeting all eligibility criteria were included in the qualitative synthesis. Figure 1 shows the flow diagram of studies analysis and selection for review.

### 2.4. Data Quality Assessment

The quality of quantitative studies and the risk of bias were assessed using the Quality Assessment Tool for Quantitative Studies (QATQS) developed within the Effective Public Health Practice Project (EPHPP) [20]. The tool was selected to provide a consistent framework for assessing the methodological quality of the quantitative evidence included in this heterogeneous review. Because the review included randomized and observational studies as well as case reports, neuroimaging studies, and preclinical studies, the applicability of the QATQS domains was considered according to the characteristics of each study design. For study types for which the tool was not specifically developed, the assessment was interpreted cautiously and was not intended to imply that the QATQS provides a design-specific estimate of risk of bias. In particular, the QATQS ratings for these studies were used as a structured assessment of methodological limitations rather than as directly comparable risk-of-bias estimates across different study designs. The tool consists of eight sections. Each section includes a set of questions addressing key methodological aspects. These include selection bias, study design, confounders, blinding, data collection methods, withdrawals and dropouts, intervention integrity, and statistical analyses. Each section is rated as 1 for high quality, 2 for moderate quality, or 3 for low quality. The global rating is based on the number of weak component scores. A study is considered high quality if no weak ratings are present. It is rated as moderate if one weak rating is identified. It is considered low quality if two or more sections receive a weak rating [21].

## 3. Results

### 3.1. Behavioral, Subjective, and Neurobiological Indicators of the Addictive Potential of MPH

Available studies consistently show that MPH can produce reinforcing effects and subjective „drug liking”. These are considered key mechanisms underlying craving. The intensity of these effects depends strongly on dose, route of administration, and pharmacokinetic profile of the drug. Behavioral self-administration models provide some of the strongest evidence that MPH can act as a reinforcer. Studies assessing MPH use have employed the progressive ratio procedure. In this paradigm, the number of responses required to obtain each subsequent dose increases progressively [22]. This allows assessment of motivational properties and abuse potential. Participants were required to perform instrumental responses in a computer-based task, such as repeated button presses. The required number of responses increased with each subsequent dose of MPH. Participants were willing to complete an increasing number of responses to obtain the drug. This was reflected in a rising break point, defined as the maximum number of responses performed to receive a dose. The highest values were observed at a dose of 32 mg. The dose–response relationship showed a bitonic pattern. This is considered a typical profile for substances with addictive potential. Similar findings were observed in choice procedures. Participants chose MPH more often than a monetary alternative. These data come from studies using the drug versus money paradigm. Fast-acting formulations showed higher reinforcing value than extended-release preparations [23]. These findings indicate that MPH can function as a strong reinforcing stimulant. The observed behaviors resemble compulsive wanting. This forms a behavioral basis for mechanisms leading to craving.

Studies using subjective measures of psychoactive effects consistently show that the reinforcing potential of MPH depends primarily on pharmacokinetics. In particular, it depends on the rate of increase in plasma concentration. Subjective effects were most often assessed using visual analog scales. These included items such as Drug Liking, High, and Take Drug Again. The latter is considered a psychometric proxy for craving. Additional measures included the Addiction Research Center Inventory. In particular, the MBG subscale reflecting euphoria and the A subscale reflecting stimulation were used [24]. Rapid attainment of peak concentration after administration of immediate-release formulations, as well as combinations of immediate-release and extended-release forms, was associated with clear euphoric effects. In contrast, extended-release formulations showed a significantly weaker and slower hedonic profile [25]. Similar results were observed in studies comparing different formulations of MPH. Immediate-release forms produced higher Drug Liking and Take Drug Again scores than controlled-release preparations. The magnitude of these effects increased with dose [26].

The most direct evidence comes from a study by Shram from 2022. In this study, d-methylphenidate was administered orally, intranasally, and intravenously. The highest Drug Liking and Take Drug Again scores were observed after intranasal and intravenous administration. These routes provide rapid delivery of the drug to the brain. The findings clearly indicate a high reinforcing potential associated with rapid increases in drug concentration [27]. These data suggest that subjective reinforcement is not an inherent property of the MPH molecule itself. It is primarily driven by the rate of increase in concentration. Fast immediate-release formulations and intranasal or intravenous administration produce pronounced hedonic responses. In contrast, extended-release formulations show minimal potential to induce subjective effects resembling craving.

Neuroimaging studies indicate that craving after MPH is not a universal response. It depends on both the pharmacokinetic profile of the drug and individual vulnerability of the reward system. In this context, craving should be understood as a subjective desire to re-administer the substance. It also involves activation of brain pathways responsible for motivation and compulsion. PET studies in non-addicted individuals have shown that expectation of MPH effects, even without actual administration, can activate the nucleus accumbens and Brodmann area 25. These structures are central to the wanting system and to the attribution of salience to rewarding stimulation [28]. A different pattern was observed in individuals with cocaine dependence. In this population, intravenously taken MPH induced craving. This effect correlated with activation of the medial orbitofrontal cortex and the anterior cingulate cortex. These regions are involved in impulsivity and maladaptive salience attribution [29]. Importantly, in healthy individuals, the same drug produced a subjective high but did not activate circuits associated with compulsive drug seeking. This suggests that craving emerges primarily in brains that have been previously sensitized.

Additional evidence comes from neuroimaging studies examining the relationship between pharmacokinetics and subjective reward value [30]. Subjective likeability of methylphenidate correlates not with the maximal level of dopamine transporter blockade, but with the rate at which it increases in the striatum. Immediate-release formulations showed rapid dopamine transporter blockade and significantly higher pleasure ratings. In contrast, OROS formulations, despite similar final levels of blockade, produced much weaker hedonic responses. These findings indicate that the speed of dopaminergic signaling, rather than its absolute magnitude, is a key neurobiological factor promoting the development of craving. A concise summary of the findings discussed in this section is presented in Table 1.

### 3.2. Tolerance to MPH

Tolerance to MPH is clinically manifested as a gradual loss of therapeutic efficacy during exposure to a constant dose, necessitating dose escalation to maintain clinical effectiveness. It may occur in an acute form, developing within hours of administration, or as a chronic process. Chronic tolerance develops over weeks to months and reflects underlying neuroadaptation. Importantly, the existence of tolerance is not inferred solely from clinical observations.

The most direct evidence of acute tolerance has been demonstrated in studies involving children with ADHD [31]. In one experimental group, pharmacokinetic profiles were manipulated to reproduce specific concentration–time curves by administering MPH and placebo capsules at 30 min intervals. Efficacy was assessed repeatedly throughout the day using SKAMP/CLAM scales and cognitive tasks. In a second group, different three-dose regimens with varying inter-dose intervals were compared, and clinical outcomes were analyzed in relation to simulated plasma concentrations and concentration–effect curves. The findings showed that the clinical effect of methylphenidate administered in the afternoon was attenuated despite sustained plasma drug concentrations, particularly under conditions of relatively constant concentration profiles. This indicates that, at the same plasma concentration, the clinical response was lower later in the day than in earlier hours. Thus, the relationship between drug concentration and clinical effect was not constant over time but depended on prior drug exposure. On concentration–effect plots, this was reflected by the formation of hysteresis loops, indicating the development of acute pharmacodynamic tolerance rather than pharmacokinetic changes. The available literature also suggests that tolerance-like phenomena may emerge even after a single exposure [13]. This was demonstrated using dynamic PET imaging assessing MPH distribution in the striatum alongside concurrent measurement of subjective “high.” A key observation was that euphoria increased rapidly and subsided within tens of minutes, whereas MPH binding in the striatum persisted considerably longer.

Chronic tolerance has been clearly demonstrated in studies assessing dopamine transporter (DAT) availability in adults with ADHD who were previously stimulant-naïve, before treatment and after 12 months of MPH therapy [32]. An important methodological element was the inclusion of a washout period sufficient to minimize the effects of acute DAT occupancy and focus on long-term changes. An increase in DAT availability in the striatum (particularly in the ventral striatum) was observed after one year of treatment. This was interpreted as a compensatory neuroadaptation to chronic DAT inhibition by MPH. Such a direction of change represents a coherent mechanism of pharmacodynamic tolerance: at the same dose and level of transporter blockade, the system may remove dopamine more efficiently during drug-free intervals.

Animal studies further support the causal chain, as they allow direct measurement of presynaptic dopaminergic markers and behavioral responses following repeated exposure [33]. Repeated methylphenidate exposure may also induce broader neuroadaptive changes extending beyond alterations in dopamine transporter function. Preclinical studies have demonstrated region-specific changes in gene expression following repeated methylphenidate administration, including alterations in immediate-early genes and neuropeptides within the striatum, consistent with drug-induced neuroadaptation [34]. Chronic methylphenidate exposure has also been associated with structural and molecular changes within corticostriatal circuits, including alterations in dendritic spine density and ΔFosB expression in nucleus accumbens neurons [35]. In addition, repeated methylphenidate administration has been shown to modify synaptic plasticity, including changes in hippocampal long-term potentiation, AMPA receptor trafficking, and AMPA-mediated synaptic responses [36]. Together, these findings suggest that repeated methylphenidate exposure produces molecular and synaptic adaptations that extend beyond acute dopaminergic signaling. However, these adaptations should not be interpreted as direct evidence of addiction, but rather as potential neurobiological substrates relevant to altered drug responsiveness, tolerance, and individual vulnerability.

Klein et al. [37] further highlighted that chronic methylphenidate exposure may be associated with broader molecular adaptations involving genes and signaling pathways related to synaptic plasticity and reward processing. Their review described alterations involving Htr7, Grik2, Zif268/Egr1, Homer1a, Arc, Cdc42, and Arp2, as well as changes in dopamine receptor and NMDA- and AMPA-receptor-related signaling. These molecular changes were identified within corticostriatal, prefrontal, and nucleus accumbens-related circuits, which are involved in synaptic plasticity, motivation, reward learning, and behavioral adaptation. Such findings provide a plausible biological framework through which repeated methylphenidate exposure could influence sensitization, reward-related learning, and individual vulnerability. However, these preclinical observations do not establish that chronic methylphenidate exposure causes addiction in humans and should therefore be interpreted as potential neurobiological mechanisms rather than evidence of a substance use disorder.

In these studies, young adult rats were repeatedly administered MPH, and subsequent behavioral responses and presynaptic dopamine neurochemistry in the striatum were evaluated, including responses to another psychostimulant (amphetamine). Repeated MPH exposure was shown to alter presynaptic dopamine regulation and behavioral responses, consistent with the development of functional tolerance and potentially cross-tolerance within DAT-related mechanisms. These findings suggest that repeated exposure may produce neuroadaptations extending beyond acute dopamine transporter blockade. In particular, preclinical evidence indicates that repeated stimulant exposure can modify molecular signaling and synaptic plasticity in a region-specific manner, potentially affecting the functional organization of corticostriatal and dopaminergic circuits. Such adaptations may represent a longer-lasting biological response to repeated methylphenidate exposure and may contribute to changes in drug sensitivity and behavioral responses. However, these neurobiological adaptations should not be equated with addiction itself, as their clinical relevance and relationship to compulsive drug use remain incompletely established. These findings complement PET observations by demonstrating that adaptation involves dopamine regulation itself, rather than merely drug pharmacokinetics.

At the clinical level, tolerance to methylphenidate is described as a phenomenon occurring in a subset of patients undergoing long-term treatment [38]. It is important to distinguish true pharmacological tolerance from secondary loss of response due to non-pharmacological factors such as misdiagnosis, comorbidities, or environmental changes. In some patients who initially achieved clinical improvement, further dose escalation of methylphenidate did not result in sustained restoration of efficacy and was associated with an increased incidence of adverse effects. Reported management strategies include temporary drug holidays and modification of dosing regimens. This clinical course is consistent with a pharmacodynamic tolerance model, in which the response to the drug diminishes with prolonged exposure. A concise summary of the findings discussed in this section is presented in Table 2.

### 3.3. MPH Withdrawal Symptoms

Withdrawal-related changes following methylphenidate (MPH) discontinuation have been reported, although they do not necessarily resemble the classical withdrawal syndrome observed with substances of dependence. In a controlled, double-blind study, discontinuation of MPH in children with ADHD did not result in the occurrence of physical withdrawal symptoms [39]. The predominant phenomenon was a rapid re-emergence of ADHD symptoms rather than the appearance of new somatic symptoms, suggesting that, at standard therapeutic doses, MPH discontinuation is generally well tolerated from a physical standpoint.

At the same time, the literature describes rare but well-documented cases of withdrawal symptoms that cannot be explained solely by symptom relapse or rebound effects. A case was reported of a 13-year-old patient who, following irregular MPH use, experienced recurrent, painful calf muscle cramps occurring exclusively on days without medication and resolving upon re-administration of methylphenidate [40]. The temporal relationship and reproducibility of symptoms suggest a withdrawal mechanism related to an abrupt decrease in dopaminergic stimulation in the nigrostriatal pathway. Similarly, a case of acute dystonia as a withdrawal symptom was described in an adult patient abusing very high doses of methylphenidate [41]. Symptoms appeared as early as the second day of detoxification and resolved following administration of an anticholinergic agent. This finding suggests abrupt dopaminergic dysregulation in the striatum after sudden cessation of chronic excessive stimulation. This case indicates that the risk of neurological withdrawal symptoms increases with both dose and duration of exposure. Additionally, a teenage patient experienced recurrent, painful priapism occurring exclusively during drug-free periods, which resolved upon re-initiation of MPH [42]. The authors proposed that abrupt discontinuation of MPH may lead to transient dysregulation of autonomic balance and dopaminergic-noradrenergic modulation, manifesting as atypical vasomotor symptoms.

The biological basis of such symptoms is supported by preclinical data [43]. Prolonged withdrawal from methylphenidate in rats led to significant adaptive changes in the dopaminergic system of the prefrontal cortex, manifested as hyperfunction of the dopamine transporter and postsynaptic hypersensitivity. These changes were not observed after short discontinuation periods but increased with the duration of withdrawal. This suggests that withdrawal symptoms may be driven by neuroadaptive mechanisms rather than solely by the loss of the drug’s pharmacological effects. A concise summary of the findings discussed in this section is presented in Table 3.

### 3.4. Misuse of MPH

Misuse of methylphenidate (MPH) constitutes a phenomenon distinct from abuse and should not be equated with it. It refers to the use of the medication outside medical recommendations, including use without a prescription, dose escalation, irregular intake, or instrumental use aimed at enhancing cognitive performance [44,45]. Misuse of methylphenidate may occur even with extended-release formulations, which, as discussed earlier, are designed to reduce abuse potential. This is illustrated by the case of a patient with diagnosed ADHD and comorbid substance use disorder, who intentionally escalated the dose of extended-release MPH to achieve subjective stimulant effects [46]. Importantly, the formulation used did not allow pharmaceutical manipulation or rapid increases in drug concentration. This suggests that the behavior was not simply a consequence of the pharmacokinetic properties of the formulation. Rather, individual vulnerability of the reward system, shaped by prior addictive experiences, played a key role.

In adolescent and young adult populations, misuse is often instrumental rather than recreational. Non-medical use of methylphenidate is more frequently motivated by the desire to improve concentration, alertness, and productivity rather than to achieve euphoria. At the same time, the risk of misuse is higher in individuals with more severe ADHD symptoms [47]. Research on ADHD diagnostics indicates that misuse of MPH may, in part, originate at the stage of disorder recognition [48]. A substantial proportion of students seeking ADHD diagnosis demonstrate non-credible effort in neuropsychological testing, leading to artificially lowered cognitive performance. Simultaneously, these individuals report elevated ADHD symptoms in self-report questionnaires despite a lack of consistent objective deficits. This pattern does not reflect global symptom fabrication but rather selective exaggeration of difficulties that align with known diagnostic criteria and increase the likelihood of diagnosis. In academic settings, access to psychostimulants, including methylphenidate, represents a significant secondary gain, as these medications are perceived as cognitive enhancers [49]. Consequently, false-positive ADHD diagnoses may occur, leading to treatment initiation in individuals without true clinical indications. Such exposure to MPH results from diagnostic processes rather than therapeutic decisions based on ADHD pathophysiology and represents a systemic source of misuse, independent of physician intent or subsequent patterns of use.

Epidemiological data confirm that misuse of methylphenidate is a prevalent phenomenon and clearly distinct from appropriate ADHD treatment [50]. Population-based studies have shown that non-medical stimulant use occurs predominantly among individuals without prescriptions and without an ADHD diagnosis [45]. It is also strongly associated with the use of alcohol, cannabis, and other psychoactive substances. Importantly, individuals using methylphenidate in accordance with medical recommendations do not exhibit an increased risk of addictive behaviors, clearly distinguishing misuse from medical use.

Clinical studies in high-risk populations indicate that misuse is concentrated within specific patient subgroups. Among individuals with ADHD, behaviors such as dose escalation or medication diversion occur almost exclusively in those with comorbid conduct disorders or substance use disorders [51]. Conversely, available evidence suggests that appropriately monitored treatment with methylphenidate in adolescents with ADHD and co-occurring substance use disorder does not increase the use of other psychoactive substances. This challenges the assumption that stimulant treatment itself leads to patterns characteristic of substance use disorders [52].

Cohort analyses further indicate that the increase in stimulant prescriptions over the past decade has not been associated with a proportional rise in compulsive use or addiction. This suggests that population-level exposure to ADHD medications does not inherently lead to increased problematic use [53]. At the same time, greater availability of stimulants increases the number of situations in which these medications may be used outside medical indications, for example, through diversion or occasional non-prescribed use. In this context, misuse of methylphenidate should be understood as a continuum phenomenon arising from the interaction of drug availability, individual neurobiological vulnerability, diagnostic practices, and social factors. It is not a simple or inevitable consequence of appropriately conducted ADHD treatment. A concise summary of the findings discussed in this section is presented in Table 4.

### 3.5. Abuse of MPH

Abuse of MPH should be defined as the intentional, non-medical use of the drug to achieve euphoric or stimulating effects. It is essential to distinguish abuse from both ADHD treatment and misuse, as abuse is directly associated with activation of the reward system and mechanisms typical of substance use disorders.

Research on abuse potential in recreational stimulant users indicates that methylphenidate, particularly in immediate-release formulations or when administered via alternative routes, can produce subjective effects similar to those of other psychostimulants. At the same time, extended-release formulations significantly reduce, but do not completely eliminate, this risk in individuals with neurobiological vulnerability [54]. The importance of route of administration and dose in the development of MPH abuse is best illustrated by clinical case reports, as they allow direct observation of how modifications in patterns of use translate into changes in clinical presentation. Under therapeutic conditions, orally administered methylphenidate at doses within the recommended range is characterized by a gradual increase in central nervous system concentration, which limits its reinforcing properties.

In contrast, a case has been described of an adult patient with diagnosed ADHD who used intranasal methylphenidate at doses far exceeding the therapeutic range [55]. This route of administration resulted in pronounced euphoric effects and the development of a compulsive pattern of use meeting criteria for stimulant use disorder. The clinical presentation did not differ substantially from that observed in cocaine abuse. A similar mechanism is illustrated by cases of parenteral methylphenidate use [56]. Bypassing the gastrointestinal tract and first-pass metabolism leads to a rapid increase in dopamine concentration within the mesolimbic system, which has been associated with acute psychiatric symptoms such as psychosis, delusional parasitosis (Ekbom syndrome) [57], and severe psychomotor agitation.

Population-level data confirm that methylphenidate abuse is not a marginal phenomenon but is concentrated within specific high-risk groups [58]. Studies based on addiction monitoring systems show that parenteral and intranasal use of MPH occurs among individuals treated for other substance use disorders, with doses far exceeding therapeutic recommendations. Similar conclusions emerge from pharmacovigilance analyses [59], which document a long-term increase in reported cases of abuse, severe psychiatric consequences, and frequent co-occurrence of polysubstance use.

A broad pharmacoepidemiological analysis demonstrates that MPH abuse primarily affects non-medical users or patients with severe substance use disorders, who often use the drug parenterally as a substitute for cocaine [60]. Only a small proportion of individuals abusing methylphenidate have a diagnosis of ADHD, clearly distinguishing the issue of abuse from appropriately conducted pharmacotherapy. These findings indicate that methylphenidate abuse is a context-dependent phenomenon, shaped by drug availability, route of administration, and neurobiological vulnerability, rather than a simple consequence of ADHD treatment. A concise summary of the findings discussed in this section is presented in Table 5.

## 4. Discussion

The results of this review indicate that MPH exhibits a measurable addictive potential. Its clinical relevance depends primarily on pharmacokinetics, route of administration, and individual vulnerability of the reward system. Beyond transient dopamine-transporter blockade, repeated methylphenidate exposure may also induce broader neurobiological adaptations within reward-related neural circuits. Repeated exposure may be associated with changes in receptor-related signaling, immediate-early gene expression, and proteins involved in postsynaptic density and synaptic organization, particularly within corticostriatal and nucleus accumbens circuits [37]. These molecular and synaptic adaptations may alter the responsiveness of neural networks following repeated stimulant exposure and may therefore contribute to the development of tolerance and changes in behavioral sensitivity. Importantly, such neurobiological adaptations should not be interpreted as direct evidence of addiction, but rather as potential mechanisms through which repeated methylphenidate exposure may produce longer-lasting changes in reward-related circuitry.

Importantly, the similarity between methylphenidate and other psychostimulants at the level of catecholaminergic neurotransmission does not necessarily imply equivalent abuse liability. The available evidence suggests that the reinforcing effects of methylphenidate are strongly dependent on the pattern and rate of exposure [23,26]. Higher doses, immediate-release formulations, and particularly intranasal or intravenous administration can produce more rapid increases in drug concentration and stronger subjective reinforcing effects than appropriately administered extended-release formulations. Therefore, the distinction between therapeutic and non-medical use cannot be explained solely by the molecular mechanism of methylphenidate. Formulation, dose, route of administration, rate of drug delivery, expectancy, and the broader context of use may all modify the reinforcing effects and the associated risk of problematic use [27,28]. This distinction is particularly important when interpreting findings from studies using non-therapeutic routes or exposure conditions, which may not be representative of appropriately monitored clinical treatment.

The collected data are consistent with previous reports suggesting that a rapid increase in dopamine concentration in the striatum constitutes a key mechanism underlying rewarding effects and craving [7], whereas the slower concentration profiles observed with oral administration of extended-release formulations significantly reduce this effect. At the same time, findings from neuroimaging studies indicate that craving is not solely a consequence of pharmacological action but also depends on cognitive processes such as reward expectation. These observations explain why, under therapeutic conditions, the risk of developing substance use disorders is relatively low, while it increases significantly in cases of non-medical use or alternative routes of administration [61]. The available evidence also suggests that pharmacodynamic tolerance may develop in some patients, whereas a typical somatic withdrawal syndrome is rare and most often limited to the recurrence of symptoms of the underlying condition.

An important limitation of the available evidence is the substantial heterogeneity of study designs, including randomized controlled trials, observational studies, numerous case reports, and preclinical models. Some conclusions regarding withdrawal symptoms and abuse are based on single clinical reports, which limits their generalizability to the broader population. In addition, many studies have assessed short-term drug effects under experimental conditions, while well-designed prospective studies in adults receiving long-term treatment remain scarce. Consequently, the strength of evidence in certain areas-particularly those concerning long-term tolerance and rare withdrawal symptoms-should be considered moderate to low. Limitations also apply to the review process itself. The analysis included publications available in English and identified through selected databases, which may have resulted in the omission of relevant studies. The inclusion of studies with heterogeneous methodologies precluded quantitative meta-analysis and increases the risk of bias related to data heterogeneity. Furthermore, publication bias cannot be excluded, as studies reporting negative or neutral findings may be underrepresented. This may contribute to an overestimation of the frequency of addiction-related outcomes. An additional limitation concerns the potential for confounding and indication bias in observational evidence. Individuals receiving methylphenidate may differ systematically from untreated individuals with respect to ADHD severity, psychiatric comorbidity, previous substance use, socioeconomic factors, healthcare utilization, and other characteristics that may independently influence the risk of substance use disorder. Conversely, patients considered to be at higher risk of problematic substance use may receive more intensive monitoring or may be preferentially prescribed particular formulations, which may further influence observed associations. Therefore, the absence of an observed increase in substance use disorder risk among treated patients should not be interpreted as definitive evidence of a causal absence of risk. Interpretation is also complicated by variability in adherence to prescribed treatment. Observational studies based on prescribing records may not accurately capture the actual pattern of methylphenidate exposure, including missed doses, dose changes, discontinuation, or non-medical use. Consequently, prescription status should not necessarily be considered equivalent to continuous therapeutic exposure. Importantly, the limited availability of long-term prospective studies restricts the ability to determine whether the absence of an observed association between therapeutic methylphenidate exposure and substance use disorder persists over prolonged treatment periods. Most available evidence addresses shorter-term effects, specific populations, or selected clinical outcomes rather than the long-term incidence of formally diagnosed substance use disorder. Accordingly, the present findings support a low observed risk under monitored therapeutic conditions, but do not establish the absence of long-term risk.

The development and magnitude of tolerance to methylphenidate may also be influenced by patient- and exposure-related factors, including dose, age, and sex. Dose should not be considered independently of treatment duration, formulation, and pharmacokinetic profile. The studies included in the present review suggest that repeated exposure may produce both short-term pharmacodynamic tolerance and longer-term neurobiological adaptation [31,32]. However, the available evidence does not establish a simple dose-dependent threshold for the development of tolerance. Age may represent an additional modifying factor. The evidence identified in this review includes acute tolerance in children with ADHD and neurobiological adaptation following longer-term exposure in adults [31,32]. Nevertheless, these studies were not designed as direct age-comparison studies, and therefore do not allow us to determine whether age independently modifies susceptibility to methylphenidate tolerance. Age-related differences should consequently be considered as a potential source of heterogeneity rather than as an established determinant of tolerance. Similarly, sex may potentially influence the pharmacokinetic and pharmacodynamic response to methylphenidate and consequently the development or magnitude of tolerance. The studies included in the present review do not provide sufficient evidence to establish a reliable sex-specific difference in methylphenidate tolerance. This represents an important gap in the current literature, particularly because direct comparisons between males and females are limited. Future studies should therefore consider sex as a potential moderator when evaluating tolerance to repeated methylphenidate exposure. Overall, the available evidence suggests that tolerance to methylphenidate is likely to be a multidimensional phenomenon influenced by dose, exposure pattern, treatment duration, pharmacokinetics, and potentially patient characteristics such as age and sex. However, the limited number of studies directly examining these interactions prevents the development of a definitive dose-, age-, or sex-dependent model of methylphenidate tolerance.

The findings of this review should be interpreted in relation to important differences between study populations and patterns of methylphenidate exposure. ADHD status, age, formulation, dose, route of administration, duration of treatment, and previous exposure to stimulants or other psychoactive substances may all influence the observed risk profile. The available literature is not sufficiently consistent to determine the independent contribution of each factor. The strongest evidence concerns formulation, dose, and route of administration, with rapid drug delivery and higher exposure being associated with greater reinforcing effects. By contrast, differences related to age, duration of treatment, and previous stimulant exposure are less well characterized because direct comparisons between these subgroups are limited. Consequently, the findings of this review should not be interpreted as applying uniformly to all patients or all patterns of methylphenidate exposure.

The findings have important clinical and systemic implications. In clinical practice, they support the preference for extended-release formulations and the avoidance of regimens that produce rapid fluctuations in drug concentration, particularly in patients at increased risk of substance use disorders. At the health policy level, they highlight the importance of monitoring medication diversion, ensuring high-quality ADHD diagnostics, and providing education on the non-medical use of stimulants. At the same time, the available data indicate that properly conducted treatment does not increase the population-level risk of addiction, which is important for rational pharmacotherapy. Future research should focus on long-term, prospective studies in adults treated with methylphenidate, incorporating standardized definitions of “addictive symptoms.” Of particular importance is the identification of biomarkers of neurobiological vulnerability to craving and tolerance, as well as clinical factors that increase the risk of transition from misuse to a full substance use disorder. Further studies comparing different drug formulations in real-world clinical settings are also needed, along with analyses of how treatment monitoring strategies may reduce diversion and non-medical use.

## 5. Conclusions

Methylphenidate demonstrates measurable reinforcing properties and may induce addiction-like symptoms. Their clinical significance depends primarily on the route of administration, the rate of increase in drug concentration, and individual neurobiological vulnerability. Under appropriately monitored therapeutic conditions, the available evidence indicates a low observed risk of methylphenidate use disorder, particularly with oral administration at recommended doses and formulations that limit rapid increases in drug concentration. However, this risk profile should be interpreted in the context of patient characteristics and exposure patterns, including age, ADHD status, previous substance-use history, formulation, dose, route of administration, and duration of treatment. The available evidence is insufficient to determine the independent contribution of each of these factors. The highest risk is observed with high doses and alternative routes of administration, which lead to a rapid increase in drug concentration within the central reward system. Available data also indicate that tolerance may develop in some patients, whereas a full withdrawal syndrome is rare. These findings highlight the importance of rational selection of drug formulation, careful treatment monitoring, and identification of patients at increased risk of problematic use.

## Figures and Tables

**Table 1:** Overview of studies evaluating the reinforcing potential of methylphenidate, with consideration of intervention type, assessment methods, and key outcomes.

Author	Study Type	Intervention	Methodology	Clinical Conclusions	QATQs
Stoops [22]	Randomized, double-blind, placebo-controlled trial	Immediate-release methylphenidate, oral: 16 mg, 32 mg, 48 mg	Behavioral measures: Progressive Ratio procedure (number of responses required to obtain a dose). Subjective measures: visual analog scales (VAS) for drug effects (“high”, “good effects”), Addiction Research Center Inventory (ARCI). Other: cognitive performance test (Digit Symbol Substitution Test), physiological parameters	The 32 mg dose significantly increased motivation for methylphenidate self-administration compared to placebo, indicating reinforcing effects and the presence of a craving component	1
Parasrampuria [23]	Randomized, double-blind, crossover trial	Immediate-release methylphenidate (50 mg, 90 mg oral) and OROS controlled-release methylphenidate (54 mg, 108 mg oral)	Subjective measures: Drug Liking, High, Take Drug Again (VAS). Psychometric scales: ARCI. Behavioral measures: Subjective Drug Value Procedure (drug vs. money)	Immediate-release formulation produced significantly greater desire for re-administration and higher reinforcing value than OROS formulation. The 54 mg OROS dose did not differ significantly from placebo	1
Jasinski [24]	Randomized, double-blind, placebo-controlled trial	Oral methylphenidate, 90 mg	Subjective measures: Drug Rating Questionnaire. Psychometric scales: ARCI (euphoria, stimulation). Behavioral measures: perceived “street value” of the drug	Methylphenidate produced strong euphoric and stimulant effects and high reported street value, recognized correlates of strong desire for repeated use	2
Spencer [25]	Randomized, double-blind, controlled trial	Immediate-release methylphenidate, extended-release methylphenidate, and their combination; oral administration	Subjective measures: VAS (“drug liking”, “high”, “want more drug”). Psychometric scales: ARCI	Rapid-onset formulations and combined immediate- and extended-release administration produced stronger rewarding effects and greater desire for re-administration compared to extended-release formulation alone	1
Bernard [26]	Randomized, double-blind, placebo-controlled trial	Oral methylphenidate, 40 mg	Psychometric scales: ARCI. Mood scales: Profile of Mood States (POMS). Performance tests: reaction time, critical flicker fusion threshold	Methylphenidate induced significant euphoric and stimulant effects in healthy volunteers, providing a behavioral basis for the development of craving with repeated use	2
Shram [27]	Randomized, double-blind, controlled trial	d-methylphenidate: oral (extended-release), intranasal, and intravenous; recreational doses adjusted to route of administration	Subjective measures: Drug Liking (Emax), Take Drug Again (VAS), Overall Drug Liking, ARCI—amphetamine scale	d-methylphenidate induced strong subjective “wanting” and desire for re-administration, particularly following intranasal and intravenous routes. Rapid plasma concentration increase was a key predictor of craving	1
Volkow [28]	Experimental study with randomized expectancy conditions using PET neuroimaging	Oral methylphenidate administered under expectancy vs. no-expectancy conditions	Subjective measures: VAS (“drug liking”, “high”, “anxiety”). Neuroimaging: FDG-PET—glucose metabolism in the nucleus accumbens and anterior cingulate cortex	Expectation of methylphenidate effects alone activated brain regions involved in motivation and craving, even in the absence of the drug. Craving mechanisms depend not only on pharmacology but also on reward prediction	2
Volkow [29]	Clinical case–control study with pharmacological challenge and PET neuroimaging	Intravenous methylphenidate: 0.50 mg/kg and 0.25 mg/kg	Subjective measures: craving assessed as “desire for cocaine” and “loss of control”. Neuroimaging: FDG-PET and dopamine receptor PET	Intravenous methylphenidate induced a significant increase in craving in cocaine-dependent individuals, correlated with activation of the striatum and orbitofrontal cortex	1
Spencer [30]	Randomized, single-dose, between-group study with PET imaging in healthy volunteers	Immediate-release methylphenidate (40 mg, oral) vs. osmotic-controlled release methylphenidate (90 mg, oral; OROS)	Twelve healthy adults were randomly assigned to receive one formulation; plasma d-methylphenidate levels were measured hourly over 10 h; dopamine transporter occupancy was assessed at 1, 3, 5, and 7 h using PET with a carbon-11-labeled ligand (Altropane); subjective effects were evaluated using the Drug Rating Questionnaire (“feel”, “like”, “dislike”); correlations between plasma levels, transporter occupancy, and subjective responses were analyzed	The osmotic-release formulation showed slower pharmacokinetics, delayed dopamine transporter occupancy, and minimal subjective likeability compared to immediate-release methylphenidate, suggesting lower abuse potential; rate of drug delivery rather than peak concentration determines abuse liability	1

**Table 2:** Characteristics of studies evaluating the development of tolerance to MPH, including applied methodology, intervention, and clinical conclusions.

Author	Study Type	Methodology	Intervention	Clinical Conclusions	QATQS
Swanson [31]	Randomized, double-blind, placebo-controlled trial	Group 1: manipulation of concentration profiles via capsule administration every 30 min; repeated within-day assessments (SKAMP/CLAM + cognitive tests). Group 2: comparison of three-dose regimens with different dosing intervals; PK/PD modeling and concentration–effect analysis	Immediate- and extended-release MPH administered in regimens reproducing different plasma concentration curves; compared with placebo	In a subset of patients, therapeutic effects diminished later in the day despite maintained plasma concentrations	1
Wang [32]	Prospective cohort study with control group (PET), non-randomized	Stimulant-naïve adults with ADHD: PET at baseline and after 12 months of treatment; healthy controls scanned at matched intervals. DAT availability measured with ROI analysis in the striatum. Follow-up scan 24 h after last dose to minimize acute DAT occupancy effects	MPH treatment for 12 months (clinically optimized doses; mean ~1 mg/kg/day)	Increased DAT availability after treatment (particularly in the ventral striatum), providing a mechanistic basis for pharmacodynamic tolerance and potential reduced efficacy at equivalent exposure	2
Sproson [33]	Preclinical animal study	Rats: repeated MPH exposure; assessment of presynaptic dopamine transmission in the striatum and behavioral changes; amphetamine challenge test	Chronic/repeated MPH administration in an animal model	Repeated MPH exposure induces presynaptic neuroadaptations and behavioral changes	2
Ross [38]	Observational study	Evaluation of management strategies (drug holidays, formulation/dosing changes, switching drug class)	MPH treatment in clinical practice	Tolerance occurs in a subset of patients. Dose escalation often fails to restore efficacy and increases adverse effects; treatment breaks and strategy modification may be beneficial	3

**Table 3:** Characteristics of studies evaluating symptoms following methylphenidate discontinuation and their clinical and neurobiological basis, including discontinuation patterns and observed outcomes.

Author	Study Type	Methodology	Methylphenidate Intervention	Clinical Conclusion	QATQs
Arnold [39]	Randomized, double-blind, placebo-controlled trial	Stabilization phase on medication followed by randomization to continuation vs. discontinuation (placebo). Assessment of symptoms and safety after treatment cessation	Abrupt discontinuation of d-methylphenidate	No evidence of a physical withdrawal syndrome or somatic “rebound” effects. The primary observation was a rapid return of ADHD symptoms following discontinuation	1
Coskun [40]	Case report	Clinical case description	MPH: previously OROS on school days and immediate-release (IR) on weekends; during summer, irregular IR 20 mg with missed doses. Symptoms occurred in the morning after drug-free days and resolved after resumption of regular IR and switch to OROS 36 mg	Painful calf cramps as a possible withdrawal symptom following irregular use; strong temporal association (after missed doses) and reversibility upon re-administration	3
Grau-López [41]	Case report	Clinical case description	MPH abuse: 600 mg/day intranasally for 2 years; during detoxification, acute dystonia occurred on day 2 after discontinuation	Acute dystonia may represent a withdrawal symptom following chronic abuse of very high doses of MPH	3
Schwartz [42]	Case report	Clinical case description	Extended-release MPH taken daily with a 24 h drug-free period on Sundays; priapism episodes occurred on drug-free days	Stuttering priapism as a rare and atypical symptom associated with withdrawal or drug-free intervals	3
Pereira [43]	Preclinical controlled study	30 days of MPH exposure followed by comparison of short (24 h) vs. long (10 days) withdrawal effects	MPH administered for 30 days; subsequent withdrawal for 24 h vs. 10 days; after 10 days, additional cocaine challenge to assess sensitization	Prolonged withdrawal (10 days), particularly in ADHD models, is associated with significant dopaminergic dysregulation (including DAT hyperfunction and postsynaptic hypersensitivity) and increased responsiveness to psychostimulants; short withdrawal (24 h) did not produce such effects	2

**Table 4:** Characteristics of studies on the non-medical use of MPH, its motivational determinants, the phenomenon of diversion, and risk factors for misuse in adolescent and adult populations, including patterns of use and clinical implications.

Author	Study Type	Participants	Methodology	Intervention	Clinical Conclusion	QATQs
Winhusen [44]	Observational study	Young adults and adults with or without ADHD	Questionnaires on stimulant use; analysis of motivations and usage patterns	Non-medical use of MPH and other stimulants	Misuse is often instrumental (focus, alertness) rather than hedonic	2
McCabe [45]	Population-based epidemiological study	Students and young adults	Large cohort samples; self-report surveys; analysis of co-occurring substance use	Non-medical use of MPH (without prescription, diversion)	Misuse mainly concerns individuals without ADHD and co-occurs with other substance use	3
Rizkallah [46]	Case report	Adult female patient with ADHD + SUD	Detailed clinical description; analysis of motivation and dose escalation	Extended-release MPH; intentional dose escalation	Misuse may occur despite ER formulations; reward system vulnerability is key, not route of administration	3
Upadhyaya [47]	Clinical-observational study	Adults and young adults with ADHD	Assessment of non-medical use and motivations for MPH use	MPH and other stimulants, partly without prescription	Misuse is more often motivated by self-medication of ADHD symptoms than by seeking euphoria	2
Sullivan [48]	Clinical-observational study	Students seeking ADHD assessment	Neuropsychological testing + effort measures; followed by validity analysis	Access to stimulants as a secondary gain	Poor effort and symptom exaggeration contribute to false-positive diagnoses and exposure to MPH	2
Suhr [49]	Clinical-observational study	Students suspected of ADHD	Assessment of malingering and symptom exaggeration	MPH as a potential secondary gain	Some ADHD diagnoses may be motivated by access to stimulants	2
Novak [50]	Epidemiological study	Young adult population	Analysis of diversion and non-medical use of MPH	MPH obtained outside the medical system	MPH misuse is primarily associated with diversion, not ADHD treatment	3
Wilens [51]	Clinical-observational study	Patients with ADHD	Analysis of patterns of use, escalation, and diversion of MPH	IR and ER MPH in ADHD treatment	Misuse risk mainly concerns patients with comorbid SUD	2
Tamm [52]	Clinical-observational study	Adolescents with ADHD and SUD	Monitored stimulant treatment	MPH under controlled conditions	Properly monitored treatment does not increase use of other substances	2
Brumbaugh [53]	Cohort analysis	General population (stimulant prescriptions)	Analysis of prescription trends and MPH use patterns	Increased availability of MPH in the population	Increased prescribing of MPH is not clearly associated with increased stimulant addiction	3

**Table 5:** Characteristics of studies evaluating abuse of MPH, including patterns of use, risk factors, and clinical implications.

Author	Study Type	Methodology	Intervention	Clinical Conclusion	QATQs
Koblan [54]	Case–control study	Analysis of pharmacodynamic mechanisms of MPH	MPH in different formulations and routes of administration	The abuse potential of MPH depends primarily on the rate of dopamine increase in the reward system	2
Coetzee [55]	Case report	Clinical description; analysis of usage pattern and addiction symptoms	Intranasal megadoses of MPH	High-dose intranasal use leads to full-blown abuse, clinically similar to cocaine abuse	3
Vérité [56]	Case report	Description of an acute psychiatric episode following parenteral MPH use	Parenteral MPH	Acute psychosis after parenteral use; high abuse potential associated with altered pharmacokinetics	3
Pereiro Gómez [57]	Case report	Clinical description of Ekbom syndrome and psychotic symptoms following MPH use	Parenteral MPH, repeated doses	MPH abuse may lead to delusional parasitosis and severe psychotic disorders, similar to other stimulants	3
Bjarnadóttir [58]	Pharmacoepidemiological study	Registry data analysis; patterns of use, routes of administration, doses	Intranasal and parenteral MPH at supratherapeutic doses	MPH abuse is concentrated in populations with prior substance use disorders	3
Guerra [59]	Pharmacovigilance analysis	Long-term analysis of adverse event reports	Various forms of MPH; often alternative routes	Increasing number of abuse reports; severe psychiatric symptoms and non-medical patterns of use predominate	3
Frauger [60]	Pharmacoepidemiological study	Comparative analysis of MPH with other stimulants	Parenteral MPH as a substitute for cocaine	MPH abuse mainly concerns recreational users; rarely patients treated according to guidelines	3

## Data Availability

No new data were created or analyzed in this study. Data sharing is not applicable.

## Supplementary Materials

The following supporting information can be downloaded at: https://zenodo.org/records/20323061, PRISMA 2020 Checklist, DOI: 10.5281/zenodo.20323061 accessed on 6 September 2026.

## References

[1] Huskamp H.A., Busch A.B., Frank R.G. (2025). Recent Trends in Medication Treatment for Attention-Deficit Hyperactivity Disorder. Psychiatr. Serv..

[2] Kim M.L., Dalvi N., Valerio D.D., Strickler G.K., Young L.D. (2023). Prescribed Stimulant Medications: Trends in the Last Decade, Pre and Post COVID-19 Response. Explor. Res. Clin. Soc. Pharm..

[3] Han B., Jones C.M., Volkow N.D., Rikard S.M., Dowell D., Einstein E.B., Guy G.P., Tomoyasu N., Ko J., Baldwin G. (2025). Prescription Stimulant Use, Misuse, and Use Disorder Among US Adults Aged 18 to 64 Years. JAMA Psychiatry.

[4] Moharram M., Kiang T. (2024). Pharmacokinetics of Long-Acting Methylphenidate: Formulation Differences, Bioequivalence, Interchangeability. Eur. J. Drug Metab. Pharmacokinet..

[5] Liu T., Gobburu J.V.S., Po M.D., McLean A., DeSousa N.J., Sallee F.R., Incledon B. (2019). Pharmacokinetics of HLD200, a Delayed-Release and Extended-Release Methylphenidate: Evaluation of Dose Proportionality, Food Effect, Multiple-Dose Modeling, and Comparative Bioavailability with Immediate-Release Methylphenidate in Healthy Adults. J. Child Adolesc. Psychopharmacol..

[6] Bieś R., Fojcik J., Warchala A., Trędzbor B., Krysta K., Piekarska-Bugiel K., Krzystanek M. (2023). The Risk of Methylphenidate Pharmacotherapy for Adults with ADHD. Pharmaceuticals.

[7] Manza P., Tomasi D., Shokri-Kojori E., Zhang R., Kroll D., Feldman D., McPherson K., Biesecker C., Dennis E., Johnson A. (2023). Neural Circuit Selective for Fast but Not Slow Dopamine Increases in Drug Reward. Nat. Commun..

[8] Rodríguez-Espinosa S., Coloma-Carmona A., Pérez-Carbonell A., Román-Quiles J.F., Carballo J.L. (2024). Tolerance, Interdose withdrawal Symptoms, and Craving Predict Prescription Opioid-Use Disorder Severity in Chronic Pain Patients: A Three-Wave Prospective Study. Psychiatry Res..

[9] Handelman K., Sumiya F. (2022). Tolerance to Stimulant Medication for Attention Deficit Hyperactivity Disorder: Literature Review and Case Report. Brain Sci..

[10] Sayette M.A. (2016). The Role of Craving in Substance Use Disorders: Theoretical and Methodological Issues. Annu. Rev. Clin. Psychol..

[11] Hu Y., Salmeron B.J., Krasnova I.N., Gu H., Lu H., Bonci A., Cadet J.L., Stein E.A., Yang Y. (2019). Compulsive Drug Use Is Associated with Imbalance of Orbitofrontal- and Prelimbic-Striatal Circuits in Punishment-Resistant Individuals. Proc. Natl. Acad. Sci. USA.

[12] Truchan J., Schepis T.S., Pasman E., McCabe V.V., McCabe S.E. (2025). DSM-5 Stimulant Use Disorder Severity, Stimulant Craving, and Other Clinical Characteristics Based on Stimulant Type (Cocaine, Methamphetamine, Nonmedical Prescription Stimulants, or Polystimulants). J. Addict. Med..

[13] Volkow N.D., Wang G., Fowler J.S., Logan J., Franceschi D., Maynard L., Ding Y., Gatley S.J., Gifford A., Zhu W. (2002). Relationship between Blockade of Dopamine Transporters by Oral Methylphenidate and the Increases in Extracellular Dopamine: Therapeutic Implications. Synapse.

[14] Volkow N.D., Ding Y.-S., Fowler J.S., Wang G.-J., Logan J., Gatley J.S., Dewey S., Ashby C., Liebermann J., Hitzemann R. (1995). Is Methylphenidate Like Cocaine?: Studies on Their Pharmacokinetics and Distribution in the Human Brain. Arch. Gen. Psychiatry.

[15] Jain R., Stark J.G. (2016). Safety and Efficacy Considerations Due to Misuse of Extended-Release Formulations of Stimulant Medications. Postgrad. Med..

[16] Kahlig K.M., Binda F., Khoshbouei H., Blakely R.D., McMahon D.G., Javitch J.A., Galli A. (2005). Amphetamine Induces Dopamine Efflux through a Dopamine Transporter Channel. Proc. Natl. Acad. Sci. USA.

[17] Faraone S.V. (2018). The Pharmacology of Amphetamine and Methylphenidate: Relevance to the Neurobiology of Attention-Deficit/Hyperactivity Disorder and Other Psychiatric Comorbidities. Neurosci. Biobehav. Rev..

[18] Grassi G., Moradei C., Cecchelli C. (2024). Long-Term Changes on Behavioral Addictions Symptoms among Adults with Attention Deficit Hyperactivity Disorder Treated with Methylphenidate. J. Behav. Addict..

[19] Page M.J., Moher D., Bossuyt P.M., Boutron I., Hoffmann T.C., Mulrow C.D., Shamseer L., Tetzlaff J.M., Akl E.A., Brennan S.E. (2021). PRISMA 2020 Explanation and Elaboration: Updated Guidance and Exemplars for Reporting Systematic Reviews. BMJ.

[20] Thomas B.H., Ciliska D., Dobbins M., Micucci S. (2004). A Process for Systematically Reviewing the Literature: Providing the Research Evidence for Public Health Nursing Interventions. Worldviews Evid. Based Nurs..

[21] Armijo-Olivo S., Stiles C.R., Hagen N.A., Biondo P.D., Cummings G.G. (2012). Assessment of Study Quality for Systematic Reviews: A Comparison of the Cochrane Collaboration Risk of Bias Tool and the Effective Public Health Practice Project Quality Assessment Tool: Methodological Research. J. Eval. Clin. Pract..

[22] Stoops W.W., Glaser P.E.A., Fillmore M.T., Rush C.R. (2004). Reinforcing, Subject-Rated, Performance and Physiological Effects of Methylphenidate and d-Amphetamine in Stimulant Abusing Humans. J. Psychopharmacol..

[23] Parasrampuria D.A., Schoedel K.A., Schuller R., Gu J., Ciccone P., Silber S.A., Sellers E.M. (2007). Assessment of Pharmacokinetics and Pharmacodynamic Effects Related to Abuse Potential of a Unique Oral Osmotic-Controlled Extended-Release Methylphenidate Formulation in Humans. J. Clin. Pharmacol..

[24] Jasinski D.R., Faries D.E., Moore R.J., Schuh L.M., Allen A.J. (2008). Abuse Liability Assessment of Atomoxetine in a Drug-Abusing Population. Drug Alcohol Depend..

[25] Spencer T.J., Spencer Biederman J., Martin J.M., Martin J.M., Moorehead T.M., Mirto T., Clarke A., Batchelder H., Faraone S.V. (2012). Importance of Pharmacokinetic Profile and Timing of Coadministration of Short– and Long–Acting Formulations of Methylphenidate on Patterns of Subjective Responses and Abuse Potential. Postgrad. Med..

[26] Bernard K., Penelaud P.-F., Mocaër E., Donazzolo Y. (2011). Absence of Psychostimulant Effects of a Supratherapeutic Dose of Tianeptine: A Placebo-Controlled Study Versus Methylphenidate in Young Healthy Volunteers. J. Clin. Psychopharmacol..

[27] Shram M.J., Setnik B., Webster L., Guenther S., Mickle T.C., Braeckman R., Kanski J., Martin A., Kelsh D., Vince B.D. (2022). Oral, Intranasal, and Intravenous Abuse Potential of Serdexmethylphenidate, a Novel Prodrug of d-Methylphenidate. Curr. Med. Res. Opin..

[28] Volkow N.D., Wang G.-J., Ma Y., Fowler J.S., Wong C., Jayne M., Telang F., Swanson J.M. (2006). Effects of Expectation on the Brain Metabolic Responses to Methylphenidate and to Its Placebo in Non-Drug Abusing Subjects. NeuroImage.

[29] Volkow N.D., Wang G.-J., Fowler J.S., Hitzemann R., Angrist B., Gatley S.J., Logan J., Ding Y.-S., Pappas N. (1999). Association of Methylphenidate-Induced Craving with Changes in Right Striato-Orbitofrontal Metabolism in Cocaine Abusers: Implications in Addiction. Am. J. Psychiatry.

[30] Spencer T.J., Biederman J., Ciccone P.E., Madras B.K., Dougherty D.D., Bonab A.A., Livni E., Parasrampuria D.A., Fischman A.J. (2006). PET Study Examining Pharmacokinetics, Detection and Likeability, and Dopamine Transporter Receptor Occupancy of Short- and Long-Acting Oral Methylphenidate. Am. J. Psychiatry.

[31] Swanson J., Gupta S., Guinta D., Flynn D., Agler D., Lerner M., Williams L., Shoulson I., Wigal S. (1999). Acute Tolerance to Methylphenidate in the Treatment of Attention Deficit Hyperactivity Disorder in Children. Clin. Pharmacol. Ther..

[32] Wang G.-J., Volkow N.D., Wigal T., Kollins S.H., Newcorn J.H., Telang F., Logan J., Jayne M., Wong C.T., Han H. (2013). Long-Term Stimulant Treatment Affects Brain Dopamine Transporter Level in Patients with Attention Deficit Hyperactive Disorder. PLoS ONE.

[33] Sproson E.J., Chantrey J., Hollis C., Marsden C.A., Fone K.C.F. (2001). Effect of Repeated Methylphenidate Administration on Presynaptic Dopamine and Behaviour in Young Adult Rats. J. Psychopharmacol..

[34] Brandon C.L., Steiner H. (2003). Repeated Methylphenidate Treatment in Adolescent Rats Alters Gene Regulation in the Striatum. Eur. J. Neurosci..

[35] Kim Y., Teylan M.A., Baron M., Sands A., Nairn A.C., Greengard P. (2009). Methylphenidate-Induced Dendritic Spine Formation and DeltaFosB Expression in Nucleus Accumbens. Proc. Natl. Acad. Sci. USA.

[36] Soares-Couto P., Sá S.I., Costa V.M., Dias-Carvalho A., Ferreira M., Carvalho F.D., Meisel A., Capela J.P. (2026). Repeated Oral Methylphenidate Administration Evokes Changes in Brain Plasticity Proteins in Juvenile Wistar Kyoto Rats: Evidence for Sex-Related Differences. Neurochem. Res..

[37] Klein S.R., Blum K., Gold M.S., Thanos P.K. (2024). Chronic Methylphenidate Effects on Brain Gene Expression: An Exploratory Review. Psychol. Res. Behav. Manag..

[38] Ross D.C., Fischhoff J., Davenport B. (2002). Treatment of ADHD When Tolerance to Methylphenidate Develops. Psychiatr. Serv..

[39] Arnold L.E., Lindsay R.L., Conners C.K., Wigal S.B., Levine A.J., Johnson D.E., West S.A., Sangal R.B., Bohan T.P., Zeldis J.B. (2004). ADouble-Blind, Placebo-Controlled Withdrawal Trial of Dexmethylphenidate Hydrochloride in Children with Attention Deficit Hyperactivity Disorder. J. Child Adolesc. Psychopharmacol..

[40] Coskun M., Kaya I. (2016). Painful Muscle Cramps Possibly Associated with Withdrawal from Methylphenidate. J. Child Adolesc. Psychopharmacol..

[41] Grau-López L., Daigre C., Mercado N., Casas M., Roncero C. (2017). Dystonia in Methylphenidate Withdrawal: A Case Report. J. Addict. Med..

[42] Schwartz R.H., Rushton H.G. (2004). Stuttering Priapism Associated with Withdrawal from Sustained-Release Methylphenidate. J. Pediatr..

[43] Dos Santos Pereira M., Sathler M.F., Valli T.D.R., Marques R.S., Ventura A.L.M., Peccinalli N.R., Fraga M.C., Manhães A.C., Kubrusly R. (2015). Long Withdrawal of Methylphenidate Induces a Differential Response of the Dopaminergic System and Increases Sensitivity to Cocaine in the Prefrontal Cortex of Spontaneously Hypertensive Rats. PLoS ONE.

[44] Winhusen T.M., Lewis D.F., Riggs P.D., Davies R.D., Adler L.A., Sonne S., Somoza E.C. (2011). Subjective Effects, Misuse, and Adverse Effects of Osmotic-Release Methylphenidate Treatment in Adolescent Substance Abusers with Attention-Deficit/Hyperactivity Disorder. J. Child Adolesc. Psychopharmacol..

[45] McCabe S.E., Teter C.J., Boyd C.J. (2004). The Use, Misuse and Diversion of Prescription Stimulants Among Middle and High School Students. Subst. Use Misuse.

[46] Rizkallah É., Legault L., Pampoulova T., Lévesque S., Bélanger M., Stavro K., Chiasson J.-P., Potvin S. (2011). A Case Report of Concerta Misuse in a Patient with Comorbid Substance Use Disorder and Attention Deficit Hyperactivity Disorder: Concerta Misuse Case Report. Am. J. Addict..

[47] Upadhyaya H.P., Kroutil L.A., Deas D., Durell T.M., Van Brunt D.L., Novak S.P. (2010). Stimulant Formulation and Motivation for Nonmedical Use of Prescription Attention-Deficit/Hyperactivity Disorder Medications in a College-Aged Population. Am. J. Addict..

[48] Sullivan B.K., May K., Galbally L. (2007). Symptom Exaggeration by College Adults in Attention-Deficit Hyperactivity Disorder and Learning Disorder Assessments. Appl. Neuropsychol..

[49] Suhr J., Hammers D., Dobbinsbuckland K., Zimak E., Hughes C. (2008). The Relationship of Malingering Test Failure to Self-Reported Symptoms and Neuropsychological Findings in Adults Referred for ADHD Evaluation. Arch. Clin. Neuropsychol..

[50] Novak S.P., Kroutil L.A., Williams R.L., Van Brunt D.L. (2007). The Nonmedical Use of Prescription ADHD Medications: Results from a National Internet Panel. Subst. Abuse Treat. Prev. Policy.

[51] Wilens T.E., Gignac M., Swezey A., Monuteaux M.C., Biederman J. (2006). Characteristics of Adolescents and Young Adults with ADHD Who Divert or Misuse Their Prescribed Medications. J. Am. Acad. Child Adolesc. Psychiatry.

[52] Tamm L., Trello-Rishel K., Riggs P., Nakonezny P.A., Acosta M., Bailey G., Winhusen T. (2013). Predictors of Treatment Response in Adolescents with Comorbid Substance Use Disorder and Attention-Deficit/Hyperactivity Disorder. J. Subst. Abuse Treat..

[53] Brumbaugh S., Tuan W.J., Scott A., Latronica J.R., Bone C. (2022). Trends in Characteristics of the Recipients of New Prescription Stimulants between Years 2010 and 2020 in the United States: An Observational Cohort Study. EClinicalMedicine.

[54] Koblan K.S., Hopkins S.C., Sarma K., Gallina N., Jin F., Levy-Cooperman N., Schoedel K.A., Loebel A. (2016). Assessment of Human Abuse Potential of Dasotraline Compared to Methylphenidate and Placebo in Recreational Stimulant Users. Drug Alcohol Depend..

[55] Coetzee M., Kaminer Y., Morales A. (2002). Megadose Intranasal Methylphenidate (Ritalin) Abuse in Adult Attention Deficit Hyperactivity Disorder. Subst. Abuse.

[56] Vérité F., Micallef J. (2017). Symptômes psychiatriques aigus lors d’injections intraveineuses de méthylphénidate: À propos d’un cas. Therapies.

[57] Pereiro Gómez C., Vicente-Alba J., Ramos-Caneda A., Vázquez Ventoso C., Fontela-Vivanco E., Díaz del Valle J.C. (2012). Ekbom syndrome in an intravenous methylphenidate abuser. Adicciones.

[58] Bjarnadottir G.D., Haraldsson H.M., Rafnar B.O., Sigurdsson E., Steingrimsson S., Johannsson M., Bragadottir H., Magnusson A. (2015). Prevalent Intravenous Abuse of Methylphenidate Among Treatment-Seeking Patients with Substance Abuse Disorders: A Descriptive Population-Based Study. J. Addict. Med..

[59] Guerra C., Soeiro T., Lacroix C., Jouve E., Micallef J., Frauger E. (2022). Augmentation de l’abus de Méthylphénidate: Repérage et Profils Sur 13 Années. Therapies.

[60] Frauger E., Amaslidou D., Spadari M., Allaria-Lapierre V., Braunstein D., Sciortino V., Thirion X., Djezzar S., Micallef J. (2016). Patterns of Methylphenidate Use and Assessment of Its Abuse among the General Population and Individuals with Drug Dependence. Eur. Addict. Res..

[61] Chang Z., Lichtenstein P., Halldner L., D’Onofrio B., Serlachius E., Fazel S., Långström N., Larsson H. (2014). Stimulant ADHD Medication and Risk for Substance Abuse. J. Child Psychol. Psychiatry.

